# Retinal Microvascular Dysfunction Occurs Early and Similarly in Mild Alzheimer’s Disease and Primary-Open Angle Glaucoma Patients

**DOI:** 10.3390/jcm11226702

**Published:** 2022-11-12

**Authors:** Stephanie Mroczkowska, Hala Shokr, Alexandra Benavente-Pérez, Anil Negi, Peter Bentham, Doina Gherghel

**Affiliations:** 1 Vascular Research Laboratory, Ophthalmic Research Group, College Health and Life Sciences, Aston University, Birmingham B4 7ET, UK; 2 Eye and Vision Research Group, School of Health Professions, Plymouth University, Plymouth PL4 8AA, UK; 3 Pharmacy Division, Faculty of Biology, Medicine and Health, University of Manchester, Manchester M13 9PL, UK; 4 Medical Innovation Development and Research Unit, University Hospitals Birmingham NHS Foundation Trust, Birmingham B15 1NT, UK; 5 Division of Cardiovascular Sciences, University of Manchester, Manchester M13 9PL, UK

**Keywords:** Alzheimer’s disease, glaucoma, retinal vessel analysis, vascular dysfunction

## Abstract

Purpose: To assess the similarities and differences in retinal microvascular function between mild Alzheimer’s disease (AD) patients, early-stage primary open angle glaucoma (POAG) patients and healthy controls. Methods: Retinal vessel reactivity to flickering light was assessed in 10 AD, 19 POAG and 20 healthy age matched control patients by means of dynamic retinal vessel analysis (DVA, IMEDOS, GmbH, Jena, Germany) according to an established protocol. All patients additionally underwent BP measurements and blood analysis for glucose and lipid metabolism markers. Results: AD and POAG patients demonstrated comparable alterations in retinal artery reactivity, in the form of an increased arterial reaction time (RT) to flicker light on the final flicker cycle (*p* = 0.009), which was not replicated by healthy controls (*p* > 0.05). Furthermore, the sequential changes in RT on progressing from flicker one to flicker three were found to differ between healthy controls and the two disease groups (*p* = 0.001). Conclusion: AD and POAG patients demonstrate comparable signs of vascular dysfunction in their retinal arteries at the early stages of their disease process. This provides support for the concept of a common underlying vascular aetiology in these two neurodegenerative diseases.

## 1. Introduction

The possibility that Alzheimer’s disease (AD) and primary open angle glaucoma (POAG) may share a common underlying aetiology has been increasingly realised over recent years [1]. This stems not only from their obvious similarities as chronic neurodegenerative diseases associated with aging, but also from the results of numerous epidemiological [2,3], pathological [4,5,6] and vascular studies [7,8,9,10].

One factor commonly attributed to the aetiology of both conditions is vascular dysfunction, particularly at the microvascular level where functional abnormalities have been suggested as initiators of the neurodegenerative disease processes in both AD [11,12,13] and glaucoma [14]. Of note is the fact that the microvascular dysfunction measured at the retinal microvascular level can also be encountered in both diseases [15,16,17,18] and, in AD, it can also correlate with the cognitive decline [19]. Moreover, when changes in retinal microvasculature were compared between AD and POAG patients, some similarities were found; however, the type of the vessels affected was different [20]. A direct comparison in the functionality of the retinal microvessels between the two disorders was, however, never performed. Increasing our understanding of the nature of any common vascular dysfunction in these conditions could, nevertheless, provide an important insight into their disease aetiologies and lead to a better awareness and understanding of their potential coexistence. Therefore, the aim of the present study was to investigate the alterations in microvascular function in AD and POAG patients at the earliest stages of their disease.

## 2. Materials and Methods

### 2.1. Patienst Recruitment

Successive, early stage newly diagnosed and previously untreated POAG patients were recruited from two local UK National Health System (NHS) Trusts. Only those patients identified as having glaucomatous cupping of the optic disc on fundoscopic assessment, normal open anterior chamber angles by gonioscopy and VF defects consistent with the diagnosis of early glaucoma using program SITA 24-2 of the Humphrey visual field analyser (HFA: Zeiss-Humphrey, San Leandro, CA) were included. For the purpose of this study an early glaucomatous VF defect was defined as a mean deviation (MD) score of <−6.00 dB [21].

Classification as POAG was based on an IOP measurements consistently above 21 mmHg on diurnal testing with applanation tonometry (measurements taken every 2 h across an 8 h period). Patients with closed iridocorneal angles, evidence of secondary glaucoma, pseudoexfoliation, history of intraocular surgery, cataract or any form of retinal or neuro-ophthalmological disease that could result in visual field defects were excluded from the study. Furthermore, newly diagnosed POAG patients with more advanced visual field loss, defined as a mean deviation (MD) score of <−6.00 dB, were also excluded from the study. All glaucoma patients were screened for cognitive impairment, using the Addenbrooke’s Cognitive Examination-Revised (ACE-R), before being included in the study.

AD patients were recruited from Birmingham & Solihull Mental Health NHS Foundation Trust (BSMHFT, Birmingham, UK) and were diagnosed with ‘probable’ AD according to the NINCDS-ADRDA criteria [22]. Patients were classified as having mild AD (Clinical Dementia Rating = 1.0 and Mini-Mental State Examination (MMSE) score 18–26) [23,24]. All AD patients were screened for glaucoma (by a glaucoma specialist- DG) before being included in the study.

Age-matched healthy controls were recruited through promotion of the study at the Aston University Health Clinics, Birmingham, UK. All healthy controls were screened for glaucoma and other ocular disease before inclusion in the study, as well as for dementia, using the ACE-R [25]. Any patients exhibiting signs consistent with glaucomatous optic neuropathy, retinal disease or who achieved an ACE-R score of less than 88 were excluded from the study [25]. Additionally, all participants were excluded if they were smokers or had a positive diagnosis of severe cardio- or cerebro-vascular disease such as coronary artery disease, heart failure, arrhythmia, stroke, transient ischemic attacks, peripheral vascular disease, severe dyslipidaemia, diabetes, as well as other metabolic disorders. Furthermore, participants with symptomatic cataract and/or cataract of grade 2.0 or above according to the lens opacities classification system III (LOCS III) [26]), were also excluded from the study due to the potential influences that cataract may have on the accurate conduction of retinal vessel analysis [27]. Well controlled systemic hypertension, defined as blood pressure readings within the normal range in those taking prescribed hypertensive medications, was neither an inclusion nor exclusion criteria for either category of patients. Any individuals taking additional medications for other chronic diseases, which could potentially further influence vascular function, were however excluded [28,29].

Ethical approval for the study was received from South Birmingham, Heart of England and Sandwell and West Birmingham NHS Research Ethics Committees, as well as the Aston University Life and Health Sciences Ethics Committee. Informed consent was obtained from all participants before entry to the study. All procedures were designed and conducted in accordance with the tenets of the Declaration of Helsinki.

### 2.2. Investigations

All measurements were performed between 8.00 a.m. and 11 a.m. following a 12 h overnight fast, which included no alcohol or caffeine.

#### 2.2.1. General Investigations

IOP was measured using Goldman applanation tonometry. Systolic blood pressure (SBP) and diastolic BP (DBP) were measured at baseline using an automatic BP monitor (UA-767, A&D Co. Ltd., Corby, UK) [30]. Ocular perfusion pressure (OPP) was then calculated as OPP = 2/3(2/3DBP + 1/3SBP)-IOP. Weight and height were recorded, and the body mass index (BMI) was calculated as: BMI = weight (kg)/height 2 (m). EDTA blood samples were obtained from the antecubital fossa vein and were tested immediately for fasting triglycerides (TGs), and total and HDL cholesterol (Total-C; HDL-C), using a Reflotron Desktop Analyser (Roche Diagnostics, Welwyn Garden City, UK). LDL cholesterol (LDL-C) was subsequently calculated as (Total-C) − (HDL-C) − (TG/5) [31,32].

#### 2.2.2. Dynamic Retinal Vessel Analysis (DVA)

Retinal vessel reactivity was measured with the dynamic retinal vessel analyser (DVA, IMEDOS GmbH, Jena, Germany). Retinal vessel diameters were recorded continuously over a 350 s time period, consisting of 50 s of baseline measurements under still illumination (25 Hz), followed by 3 cycles of 20 s flicker stimulation (optoelectronically generated at 12.5 Hz) each interrupted by 80 s of still illumination (recovery). All measurements were performed in a quiet, temperature-controlled room (22 °C) following full dilation of one pupil (1% tropicamide, Chauvin Pharmaceuticals Ltd., Kingston Upon Thames, UK) and were taken from the inferior temporal vessel branches approximately one and a half disc diameters from the optic nerve head. For POAG patients, all measurements were conducted in the eye with the greatest degree of mild glaucomatous damage, as indicated by visual field MD score and in accordance with the previously stated inclusion/exclusion criteria.

**Figure 1:**
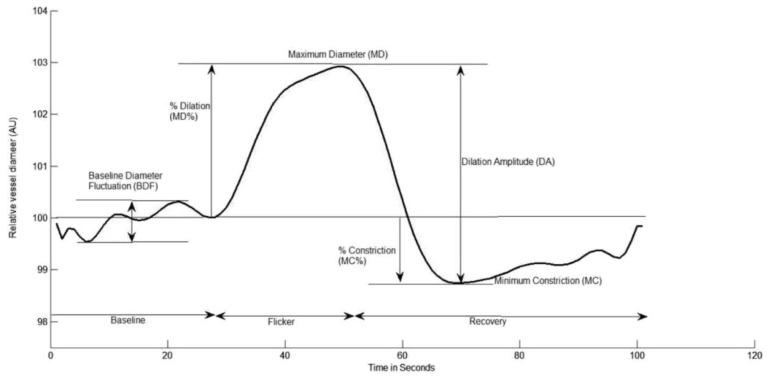
Graphical presentation of the dynamic vessel response profile displaying the parameters calculated and used in analysis. (DA) calculated as (MD-MC). (MD%) calculated as the percent increase from baseline to MD. (MC%) calculated as the percent constriction below baseline following MD.

Figure 1 depicts the dilation and constriction parameters used for analysis. In addition, baseline corrected flicker response (BFR), a parameter which indicates the overall dilation response of the vessels to flicker after normalising for the fluctuations in baseline diameter which occur with arterial pulse [33]), was also calculated in all cases by subtracting the baseline diameter fluctuation (BDF) from the difference between the maximum diameter (MD) and maximum constriction (MC).

### 2.3. Statistical Analysis

All data were reported as mean ± standard deviation. The Kolmogorov–Smirnov test was used to determine the distribution of the data. Multivariate analysis was performed to determine the influence of age, BMI, BP and circulating markers on the measured variables. Differences between groups were subsequently assessed using one-way ANOVA or ANCOVA, as appropriate, followed by Tukey’s post hoc analysis. Two factor repeated-measures ANOVA was used to compare the retinal reactivity responses across each flicker cycle. In cases where the normality of the data could not be confirmed log transformations were made. *p*-values of less than 0.05 were considered significant, except in certain cases where a stricter *p*-value of less than 0.01 was adopted in order to correct for multiple comparisons. All analyses were performed using Satistica, version 12.0, Statsoft, Tusla, OK, USA.

## 3. Results

10 mild AD patients, 19 POAG patients and 20 healthy controls were recruited for this study. There were no significant differences in age, systemic BP, BMI, triglycerides, glucose, HDL, LDL and total cholesterol levels between the groups (all *p* > 0.05, ANOVA, Table 1). Furthermore, the number of subjects with well controlled high BP was proportionally similar between groups (AD: *n* = 3; POAG: *n* = 3; Controls: *n* = 6; Chi square test, *p* = 0.530). As expected IOP was found to be significantly greater in our POAG patients in comparison to AD and healthy control groups (ANOVA, *p* < 0.001, Table 1) and OPP was subsequently found to be lower (ANOVA, *p* < 0.001, Table 1).

### 3.1. Dynamic Retinal Vessel Analysis

For ease of interpretation, the dynamic retinal vessel profile curve was considered in two parts, the first part being the dilation response (baseline to maximum dilation) and the second part being the constriction response (maximum dilation to maximum constriction). The flicker cycles were analysed both on average and individually using traditional ‘sequential and diameter response’ (SDRA) analysis, with the artery and vein being considered separately [34,35,36,37].

#### 3.1.1. Arterial Response

No significant differences were found in the average maximum diameter (MD%), reaction time (RT), baseline corrected flicker response (BFR), maximum arterial constriction (MC%) or the time taken to reach maximum constriction (tMC) between all study groups (ANOVA, all *p* > 0.01, Table 2). When considering each flicker cycle individually however, the arterial RT was found to be significantly longer on the final flicker cycle (F3) in both AD and POAG patients in comparison to healthy controls (ANOVA, *p* = 0.009, Table 3). Furthermore, the sequential changes in the RT of the retinal arteries on progressing from flicker 1 to flicker 3 was found to vary significantly between groups (ANOVA, *p* = 0.001, Table 3), with healthy controls showing a significant decrease in RT on going from F2 to F3 (ANOVA, *p* = 0.011, Table 3) which was not replicated by any of the other groups, where in fact an increase was observed.

#### 3.1.2. Venous Response

No significant differences were found in the maximum venous diameter (MD%), reaction time (RT), baseline corrected flicker response (BFR), maximum constriction (MC%) or the time taken to reach maximum venous constriction (tMC) between all study groups (all *p* > 0.01, ANOVA, Table 4).

## 4. Discussion

This study has revealed for the first-time evidence of altered reactivity to flicker light in the retinal arteries of both, previously untreated, early stage POAG patients and mild AD patients. These findings were found to be similar in both types of patients (POAG and AD) and were not replicated by the healthy controls’ group. Indeed, the time taken for the retinal arteries to reach the point of maximum dilation following the onset of flicker light stimulation was found to be significantly greater in both AD and POAG patients in comparison to healthy controls, on the final flicker cycle. Furthermore, the sequential changes in the RT of the retinal arteries on progressing from flicker 1 to flicker 3 was found to vary significantly between all study groups with healthy controls showing a significant faster RTs on heading into the final flicker cycle, which was not replicated by any of the diseased groups.

The concept that AD and glaucoma may share a common underlying vascular aetiology has been increasingly investigated over recent years, and evidence of vascular dysfunction that was related to either disturbed vascular autoregulation or disturbed neurovascular coupling mechanisms, has been previously demonstrated at the cerebral [38,39,40] and ocular level [41,42] in both conditions, but separately. Nevertheless, a direct comparison of various microvascular function parameters between these two conditions has never been made. The present study addresses this gap in the knowledge and, using the assessment of retinal vessels functionality, was able to demonstrate the presence of a similar vascular dysfunction in both newly diagnosed AD and POAG patients. Although the exact cause of these findings is currently unclear, we can hypothesise that, as the retinal vascular response to flickering light is predominantly a neurovascular coupling driven response [43,44] the significantly prolonged arterial RT demonstrated here by both patient groups could be the result of a disturbance at this level, which is further exacerbated and emphasized by the stress induced by repeated flicker stimulation [32,33,34,45]. Leading on from this, the variability in the progression of the arterial RT over successive flicker cycles and the significant involvement of the final flicker cycle in our POAG and AD patients could suggest that exhaustive factors, such as a progressive depletion of nitric oxide (NO) after repeated induced vasodilation cycles, could also play a role [46,47]. Such a depletion of NO levels could additionally be attributed to altered astrocyte activity or high levels of oxidative stress in AD and POAG [48,49,50] both of which are closely linked to endothelial dysfunction [50,51,52].

Other factors such as cholinergic receptor degeneration [53,54] or the deposition of beta-amyloid with subsequent impairment of neuronal NO production can also be implicated [55]. Indeed, the presence of microvascular dysfunction, in the form of altered neurovascular coupling responses and disturbed NO activity such as that hypothesised here, has been previously linked to the occurrence of neuronal ischemia and reperfusion injury in both POAG and AD [56,57,58].Therefore, it is possible that persistent episodes of neuronal ischemia, resulting from the presence of microvascular dysfunction and occurring whenever metabolic demand is high, could potentially lead to the development of chronic tissue hypoperfusion and subsequently to the development of neurodegeneration at either the optic nerve or cerebral level in POAG and AD [59,60]. Further investigation is obviously required however to validate all of these hypotheses before any firm conclusions can be made.

The main limitation of our study consists of its small sample. However, this has been dictated by the need of having patients free of other pathologies or concomitant medication that could represent confounding factors for our results. Nevertheless, this approach could also be considered a strength as it offered us the possibility to report clean data and, as such, true similarities between these two neurodegerative disorders in functional abnormalities assessed at the retinal vessels.

## 5. Conclusions

Our research shows for the first time that retinal microvascular abnormalities are present similarly in AD and POAG patients. As these changes can represent signs of increased risk for future deterioration their detection at early stages of the neurodegenerative process, could allow individual tailored preventive measured that improve vascular function and ultimately, the patients’ prognosis. Such a finding has potential relevance for the early diagnosis and management of both conditions, especially as microvascular abnormalities are thought to develop at the earliest stages of a disease process, prior to the onset of cognitive impairment in AD and prior to the onset of optic nerve damage in POAG.

## Figures and Tables

**Table 1:** Summary of systemic characteristics of the study groups.

	AD (1)	POAG (2)	Controls (3)	ANOVA *p*-Value	Significance
**N**	10	19	20	-	-
**Gender**	5F:5M	9F:10M	8F:12M	0.737	-
**Age (years)**	62.50 ± 8.07	63.93 ± 8.26	58.00 ± 4.32	0.079	-
**SBP (mmHg)**	141.70 ± 14.21	135.50 ± 16.92	131.70 ± 17.90	0.318	-
**DBP (mmHg)**	80.30 ± 7.51	78.86 ± 10.40	79.70 ± 9.39	0.930	-
**BMI**	27.61 ± 5.80	27.36 ± 4.03	27.56 ± 4.67	0.990	-
**Glucose**	4.40 ± 1.44	4.44 ± 1.03	4.87 ± 1.02	0.469	-
**TG**	1.28 ± 0.60	1.09 ± 0.35	1.17 ± 0.40	0.575	-
**HDL-C (mmol/L)**	1.33 ± 0.25	1.23 ± 0.24	1.14 ± 0.32	0.245	-
**Total-C (mmol/L)**	4.77 ± 0.64	4.20 ± 0.84	4.73 ± 0.67	0.089	-
**LDL-C**	3.18 ± 0.67	2.87 ± 0.75	3.38 ± 0.74	0.368	
**IOP (mmHg)**	16.50 ± 2.12	23.25 ± 2.38	17.20 ± 2.68	<0.001 *	2 > 1, 3; 1 = 3
**OPP**	84.96 ± 9.46	47.12 ± 16.93	82.65 ± 12.06	<0.001 *	2 < 1, 3; 1 = 3

Abbreviations: AD: Alzheimer’s disease; POAG: primary open angle glaucoma; ANOVA: analysis of variance; N: number; SBP: systolic blood pressure; DBP: diastolic blood pressure; BMI: Body mass index; TG: triglycerides; HDL-C: High density lipoprotein cholesterol; Total-C: Total cholesterol; LDL-C: low density lipoprotein cholesterol; IOP: Intraocular pressure; OPP: ocular perfusion pressure. * *p* < 0.05 is considered a significant difference.

**Table 2:** Average Arterial Vascular Function Parameters Determined Using Dynamic Retinal Vessel Analysis.

ARTERY Average Data	AD (1)	POAG (2)	Controls (3)	*p*-Value
MD (%)	5.53 ± 3.25	5.75 ± 3.57	5.19 ± 2.19	0.853
RT	24.04 ± 11.74	21.58 ± 6.89	20.48 ± 6.77	0.442
BFR	3.39 ± 3.79	2.55 ± 4.58	2.80 ± 1.89	0.832
MC (%)	−3.23 ± 1.56	−4.58 ± 4.58	−2.55 ± 1.85	0.100
tMC (secs)	29.00 ± 10.24	33.47 ± 10.07	26.43 ± 8.29	0.100

Abbreviations: MD (%): percentage change in diameter from baseline to maximum; RT: reaction time BFR: baseline corrected flicker response MC (%): percentage constriction below baseline; tMC: time taken to reach maximum constriction.

**Table 3:** Arterial Reaction Time by Flicker Cycle.

ARTERY	AD (1)	POAG (2)	Controls (3)	*p*-Value	Significance	Between Groups *p*-Value
**RT**						
Flicker 1	29.30 ± 16.61	18.67 ± 13.16	20.05 ± 12.07	0.134		
Flicker 2	16.30 ± 11.48	20.60 ± 12.12	25.85 ± 9.00	0.068		
Flicker 3	27.89 ± 17.62	27.92 ± 10.14	15.55 ± 11.07	0.009 *	1, 2 > 3	
**Within groups ANOVA**	0.093	0.067	0.011 *			0.001 *

Abbreviations: *p* < 0.05 (*) is considered as significant on repeated measures ANOVA. RT: reaction time.

**Table 4:** Venous Vascular Function Parameters Determined Using Dynamic Retinal Vessel Analysis.

VEIN	AD (1)	POAG (2)	Controls (4)	ANOVA *p*-Value
MD (%)	6.12 ± 3.14	5.24 ± 1.53	5.13 ± 2.86	0.614
RT	22.67 ± 9.39	19.69 ± 4.24	20.48 ± 3.68	0.430
BFR	2.16 ± 4.03	2.83 ± 2.54	3.30 ± 2.28	0.595
MC (%)	−2.61 ± 2.13	−2.54 ± 2.45	−1.77 ± 1.40	0.422
tMC (secs)	29.74 ± 6.20	34.10 ± 8.40	34.25 ± 9.40	0.443

Abbreviations: MD (%): percentage change in diameter from baseline to maximum; RT: reaction time BFR: baseline corrected flicker response MC (%): percentage constriction below baseline; tMC: time taken to reach maximum constriction.

## Data Availability

The data presented in this study are available on request from the corresponding author. The data are not publicly available due to ethical regulations.
